# The “new normal” of hygiene measures at the end of the COVID-19 epidemic: a survey among French dentists

**DOI:** 10.1186/s12913-023-10167-6

**Published:** 2023-11-03

**Authors:** Charles Broyer, Gabriel Fernandez de Grado, Damien Offner

**Affiliations:** 1 Faculté de Chirurgie Dentaire, Université de Strasbourg, 8 rue Ste Elisabeth, Strasbourg, F-67000 France; 2 Pôle de Médecine et Chirurgie Bucco-Dentaires, Hôpitaux Universitaires de Strasbourg (HUS), 1 Place de l’Hôpital, Strasbourg, F-67000 France; 3 INSERM (French National Institute of Health and Medical Research), UMR 1260, Regenerative Nanomedicine (RNM), FMTS, Strasbourg, F-67000 France

**Keywords:** COVID-19, Hygiene, Dentistry, Personal protective equipment (PPE), Predictive approach, Disease prevention

## Abstract

**Objectives:**

The COVID-19 epidemic upset the standards in terms of hygiene and protection in the dental office, bringing additional precautions for dentists. The objective of our study was to draw the “new normal” of hygiene measures at the end of the COVID-19 epidemic.

**Materials and methods:**

A self-administered questionnaire about transitional recommendations for oral care in the context of the COVID-19 epidemic was published online in private groups dedicated to French dentists.

**Results:**

The 246 respondents understood the reasons behind those recommendations, since 10 out of 11 measures reached a mean score greater than 2.5 on a 0 (not at all) to 4 (absolutely) scale when it came to determining whether the measure made the practitioner feel safe and ensured patient safety. Besides, more of the respondents intended to maintain the measures than they were to apply them before the epidemic.

**Conclusions:**

The COVID-19 epidemic reshaped the relationship to hygiene and protection measures in the context of dental practices. The “new normal” of hygiene measures at the end of the COVID-19 epidemic will probably involve more protective measures than before.

**Clinical relevance:**

These results constitute interesting avenues for public health deliberation, which would make it possible to best adapt future health recommendations in order to define the “new normal” of hygiene measures in dental practices at the end of the COVID-19 epidemic. Therefore, it could have an impact on all practitioners in their clinical activities.

## Introduction

At the end of 2019, a new coronavirus, SARS-CoV-2, was discovered in Wuhan (China) [1]. It is highly contagious, infects humans, and is the cause of the Coronavirus disease 2019 (COVID-19) [2]. It spread so much across the world, that the World Health Organization (WHO) declared the outbreak an “international public health emergency” on January 30, 2020 [3]. Transmission of SARS-CoV-2 occurs from person to person through respiratory micro-droplets by vocalization and to a greater extent when the infected subject coughs or sneezes [4]. Therefore, the production of aerosols during dental procedures represents a real risk of infection in the exercise of the profession [5, 6], because the particles suspended in the air can persist for more than 3 h [4, 7]. Even if well-established healthcare hygiene and safety habits within the profession seemed to limit this risk [8], additional measures were expected by and from dentists to best protect themselves and patients. Meanwhile, in France, the closure of dental offices was decided by the National Council of the Order of Dentists (Ordre National des Chirurgiens-Dentistes - ONCD) between March 16 and 19, 2020 [9].

On March 16, 2020, the National College of University Dentists in Public Health (Collège National des Chirurgiens-Dentistes Universitaires en Santé Publique - CNCDUSP) published the “Risks and recommendations for oral care in the context of the Coronavirus epidemic” [10]. This initial document was widely consulted, taken up and supplemented with the scientific data known later, by the French General Directorate of Health (Direction Générale de la Santé – DGS), the ONCD [11] and the French High Authority for Health (Haute Autorité de Santé – HAS) [12]. It finally ended up in a series of additional measures qualified as “transitional”, shared on July 15, 2020 [11] and still in effect to date (part of these measures are presented in Table 1). In dental clinics, these infection control measures were for example the use of adequate personal protective equipment (PPE, for example the use of FFP2 mask during aerosol generating procedures), a room aeration between patients, the reinforcement of disinfection procedures, an increased waiting time between patients to allow for these infection control measures and aeration, or the use of a dental dam whenever it is possible [13, 14]. A recent french study [13] and international data showed that dentists were not overexposed to COVID-19 when these adequate preventive measures were applied [13, 15]. Indeed, odds of COVID-19 were lower in dentists wearing FFP2 (or equivalent (K)N95) masks during aerosol or non-aerosol generating procedures [13].

**Table 1:** Answers to the survey from the 246 participant dentists. (* : *p-value* < 0.01 ; ** : *p-value* < 0.001)

Measure	Criteria	0	1	2	3	4	Mean score	Yes	No	*p*
Clear the waiting room of any object (book, magazine, toy, etc.)	Practitioner feels safe	21	26	41	54	103	2.8			
	Feeling of safety for patients	5	17	24	75	124	3.2			
	Already applied before COVID-19							38	208	**
	Intention to maintain							147	99	
Clear the surfaces around the dental unit likely to receive projections	Practitioner feels safe	7	6	43	65	124	3.2			
	Feeling of safety for patients	4	5	30	70	136	3.1			
	Already applied before COVID-19							141	105	**
	Intention to maintain							210	36	
Inform patients that they must wear a facemask when arriving	Practitioner feels safe	11	9	31	55	139	3.2			
	Feeling of safety for patients	5	5	24	54	157	3.4			
	Already applied before COVID-19							7	239	**
	Intention to maintain							39	207	
Avoid physical contact (shaking hands for example)	Practitioner feels safe	8	4	23	59	152	3.4			
	Feeling of safety for patients	8	8	26	45	159	3.4			
	Already applied before COVID-19							56	190	**
	Intention to maintain							181	65	
Only welcome the patient to be treated (1 accompanying person for minors and non-autonomous patients)	Practitioner feels safe	15	6	43	63	119	3.1			
	Feeling of safety for patients	14	12	46	48	126	3.1			
	Already applied before COVID-19							59	187	**
	Intention to maintain							161	85	
wear an FFP2 mask during aerosol treatments	Practitioner feels safe	5	3	11	57	170	3.6			
	Feeling of safety for patients	14	7	21	55	149	3.3			
	Already applied before COVID-19							8	238	**
	Intention to maintain							145	101	
Wear protective glasses during aerosol treatments	Practitioner feels safe	4	6	18	52	166	3.5			
	Feeling of safety for patients	35	17	38	39	117	2.8			
	Already applied before COVID-19							184	62	*
	Intention to maintain							210	36	
Wear protective clothing (apron or overcoat) during aerosol treatment	Practitioner feels safe	26	15	41	68	96	2.8			
	Feeling of safety for patients	34	23	41	56	92	2.6			
	Already applied before COVID-19							4	242	**
	Intention to maintain							88	158	
Favor the use of a dental dam in all possible clinical situations	Practitioner feels safe	34	16	53	62	81	2.6			
	Feeling of safety for patients	34	15	60	46	92	2.6			
	Already applied before COVID-19							80	166	*
	Intention to maintain							109	137	
Ventilate the treatment room at least 15 min after an aerosol treatment	Practitioner feels safe	8	3	28	70	124	3.1			
	Feeling of safety for patients	5	2	29	55	141	3.2			
	Already applied before COVID-19							37	195	**
	Intention to maintain							145	86	
Avoid using air conditioning while aerosols are being produced or suspended	Practitioner feels safe	30	15	72	33	38	1.7			
	Feeling of safety for patients	27	17	67	36	41	1.7			
	Already applied before COVID-19							6	181	**
	Intention to maintain							37	151	

In other places than France, recommendations flourished as well. Experts in dentistry and hygiene from several countries (USA, Great Britain, Central America, Poland, etc. among other countries) worked on recommendations to apply in their own country, because the COVID-19 epidemic had quite the same impact on the dental healthcare worldwide. This resulted in a body of recommendations, which ultimately overlapped on measures to be implemented. Like the French recommendations, we could find: room aeration, wear of adequate PPE, increased use of dental dam, mitigation of aerosol-generating procedures, etc. [16–20].

However, with time and the help of vaccines [21], the epidemic seems to slowly be running out of steam in France. Moreover, general and specifically orofacial adverse effects from widely used COVID-19 vaccines were reported to be rare [22]. The objective of our study was to determine whether the epidemic and the transitional hygiene measures had an impact on the practice of French dentists. Indeed, will they have become more aware and cautious of the infectious risk in the exercise of their profession, or will they return to the initial measures? What will be the “new normal” of hygiene measures at the end of the COVID-19 epidemic, namely the measures that the majority of dentists plan to follow?

## Materials and methods

### Study design and recruitment

We used a Google© form questionnaire (Google, Mountain View, USA) that was online from March 31, 2021 to April 19, 2021. The link to this questionnaire was shared on the social media Facebook (https://www.facebook.com) in two private groups (i.e. with restricted admission) dedicated to French dentists, either with or without specializations, with a total of 5200 members. It was a voluntary study. Data collection was anonymous, and neither name nor personal information such as email address was collected. Participants were informed of the study’s goal, the anonymous participation, and the absence of consequences in case of non-participation in the study. We complied with the French Data Protection Agency CNIL’s Reference Methodology (MR-003) for the data collection, preservation and protection.

### Questionnaire

The self-administrated questionnaire was divided into two parts. The first part was related to general information (i.e., gender, age, type of practice – dental practice, in group or alone, hospital practice, … –), the second part focused on specific measures of the “transitional recommendations” for oral care in the context of the COVID-19 epidemic [11] and studied systematically:

- if this measure made the practitioner feel safe (on a 0–4 scale : 0 for “not at all”, 4 for “absolutely”).
- if this measure gave the practitioner the feeling of guaranteeing the safety of his/her patients (on a 0–4 scale : 0 for “not at all”, 4 for “absolutely”).
- if the practitioner already applied this measure before the COVID-19 epidemic (yes/no).
- if the practitioner intended to maintain this measure regardless of the sanitary evolution (yes/no).

This original questionnaire was developed by the department of oral public health, faculty of dentistry of Strasbourg, France. Its construction was based on an internal working group, considering the objectives of the study, and largely exploring the “transitional recommendations” for oral care in the context of the COVID-19 epidemic [11]. Its content validity was established through a pilot testing: it was previously sent to 10 general dental practitioners selected randomly in the geographical region of the faculty, 2 hospital and university practitioners, and 3 national experts in the field of hygiene measures. Their answers allowed us to adjust the questionnaire and make sure the questions were clear, unbiased, and free from leading or loaded language. The questionnaire was deliberately systematized, using the same pattern for each question, only changing the specific hygiene measure of the recommendations that was analyzed, by repeating the formulation written in the official document [11]. We chose to use a Likert scale from 0 to 4 because it allowed to reliably measure the opinion of practitioners, with a minimum of ambiguity, and because it provided precise data, easy to interpret and transform into statistics [23]. Each item was analyzed as a categorical variable with 5 options (0 to 4), as visible in Table 1. The values were then processed by calculating the mean score of each specific hygiene measures regarding the studied item (feeling of own safety / safety of patients), and comparison were made whether practitioners intended to maintain or not each measure.

### Statistical analysis

Statistical analysis was performed using the BiostaTGV online platform (https://marne.u707.jussieu.fr/biostatgv/) developed by INSERM (“Institut National de la Santé et de la Recherche Médicale”, Paris, France). Chi-square tests were used to compare the proportions between two groups or more. Excel software (Microsoft Office) was used to classify and analyze the data collection. A p-value lower than 0.05 was considered statistically significant.

## Results

A total of 246 dentists answered the questionnaire, representing a response rate of 4.73%. Participating subjects were primarily women (65.4%; n = 161). The majority of respondents were under the age of 46 (68.3%; n = 168) and practiced in a private practice (85.7%; n = 211), mostly in group (58.5%; n = 144) (Table 2).

**Table 2:** Demographic and work characteristics of the 246 respondents

	*n* (N = 246)	% of the population
Sex		
Women	161	65.4
Men	85	34.6
Age		
18–30	82	33.3
31–45	86	35
46–60	61	24.8
Over 60	17	6.9
Type of practice		
Private practice, in group	144	58.5
Private practice, alone	67	27.2
Hospital practice	4	1.6
Mixed hospital and private practice	18	7.3
Helathcare center	13	5.3
Other	0	0

All the studied measures of the “transitional recommendations” reached a mean score greater than 2.5 on the 0 to 4 scale except one (“Avoid using air conditioning while aerosols are being produced or suspended”), both for the feeling of safety for the practitioner and for the feeling of guaranteeing the safety of patients (Table 1). Depending on the studied measure, more or less practitioners already applied the measure before the COVID-19 epidemic, or intended to maintain it regardless of the sanitary evolution. However, in all cases without exception, more of them intended to maintain the measures than they were to apply them before the epidemic, with statistically significant results (Table 1).

Cross-sectional analyzes showed very similar results, whether by sex, age or type of practice. No difference was statistically significant.

## Discussion

### Sustainability of hygiene measures

From a review of the scientific literature, our study was the first to explore the future implementation of hygiene measures for the post-COVID-19-epidemic period. The transitional recommendations for oral care in the context of the COVID-19 epidemic [11] have been well understood and accepted by dentists. Indeed, most of the measures reached a mean score greater than 3 on the 0 to 4 scale, meaning that dentists felt safe, for themselves and for their patients, when applying these measures. Moreover, when analyzing the intentions to maintain some of these measures, it appeared that there will probably be a *before* and an *after* COVID-19 epidemic concerning the hygiene and safety measures in dental practices. A large majority of dentists intended to maintain most of these measures, regardless of the sanitary evolution. We can nevertheless put this into perspective, because during the survey, the epidemic was even more active than today. Perhaps behaviors would be more permissive as the epidemic wanes.

Looking more closely, we can differentiate three trends in the reception of these measures. (1) First, the frankly accepted measures: the practitioners gratified them as safe and were inclined to maintain them in the future. For example: *Clear the surfaces around the dental unit likely to receive projections, Avoid physical contact (shaking hands for example), Only welcome the patient to be treated (1 accompanying person for minors and non-autonomous patients), Wear protective glasses during aerosol treatments*. Those were also part of the cheapest measures to apply, the measures that were well applied before the COVID-19 epidemic, and part of those that do not compromise the dentist’s comfort. (2) Second, the measures accepted more timidly, little applied before but which were perceived as an improvement in the care of patients and tend to be maintained. For example: *Clear the waiting room of any object (book, magazine, toy, etc.), wear an FFP2 mask during aerosol treatments, Ventilate the treatment room at least 15 min after an aerosol treatment*. Even if they have an impact on the practitioner’s comfort and on his/her profitability (15-minutes ventilation after an aerosol treatment without patient), these measures will probably be maintained by most practitioners who perceived them as an increased safety against infections. (3) Finally, measures that had a very questionable reception. Even if more practitioners wanted to keep them than those who already applied them, they were still few in favor of these measures. For example: *Inform patients that they must wear a facemask when arriving, Wear protective clothing (apron or overcoat) during aerosol treatment, Avoid using air conditioning while aerosols are being produced or suspended*, and surprisingly *Favor the use of a dental dam in all possible clinical situations*, although it has already been documented that dental dams are less used than one might wish [24, 25]. Some authors suggested that this could be due to the fact that dentists may not be aware of its effects on reducing aerosol production, or that dentists are less willing to use it due to a perceived difficulty to use it [26]. In our study, these measures globally had lower scores than others; they were also part of the measures that could impact the practitioner’s comfort.

Other studies compared the application of infection control measures before and after the COVID-19 crisis. Nevertheless, these studies were undertaken just after the crisis, during spring or summer 2020, when COVID-19 was still highly active in our everyday life. In our questionnaire, we specified that we focused on the answers regardless of the sanitary evolution. These studies reported significant increases in the frequency of use of PPE by dentists before and after the COVID-19 crisis, for example: from 0% of dentists before to 46.5% after the COVID-19 crisis concerning the use of N95 masks (equivalent of FFP2); from 44.1% before to 1.6% after who did not wear glasses or face shield during dental procedures [26]. These measures were well received in our study as well, and the same trend was observed in a french study of 2020 [13]. Another measure was the use of dental dam in every possible situation: in the same previous study [26], there was not much difference before (1.6%) and after (4.7%) the COVID-19 crisis. In our study, this measure had a questionable reception from dentists as well. Another point of vigilance is the translation of knowledge into practice when it comes to the use of PPE. A recent study pointed out that there is a need for PPE training and adherence to doffing protocols, because common errors have been highlighted and could jeopardize the safety of using PPE [27].

Some authors wondered on the possible negative effects of reinforced hygiene measures [28]. Indeed, as described by the hygiene hypothesis [29], these measures could reduce the immunity of the population because contact with pathogens would be minimized. However, these measures were already partially applied in the dental office, and do not transpose to the everyday life of patients: it is only a question of an environment of care. In accordance with the Hippocratic “first, do no harm” (*primum non nocere*) and because it is the responsibility of the practitioner, the patient should be protected during the care activity. This does not prevent contact with pathogens in other circumstances so as not to lose our immunity, for example but not only through the vaccines that have been developed [21].

### Lessons from COVID-19 pandemics and recommendations for more predictive and preventive approach in the future

Since the transitional recommendations for oral care in the context of the COVID-19 epidemic [11] have been well understood and accepted by dentists, we can assume that some lessons have been learned from COVID-19 pandemics. Therefore, in addition to hygiene measures, it is still important to engage evolutions in health policies [30]. Indeed, there is a need to respond to future pandemics more time-efficiently and cost-effectively [31], and all the more so in oral public health because new viruses or pathogens could be linked to oral health. For example, it has been showed that a poor oral hygiene and a poor oral health can be linked to an aggravation of COVID-19 symptoms [32]. Predictive, preventive and personalized medicine (PPPM) could be an interesting way to move from reactive healthcare services to preventive ones [30], while developing methods and tools to enhance the accuracy of predictive diagnosis and favoriting targeted prevention [33]. These methods could be, for example, the increased use of artificial intelligence and rapid diagnosis [30]. PPPM could therefore lead, *via* more efficient prevention, to a reduction of dental procedures, and logically to a reduction of aerosols and risks in dental practices. This would meet many recommendations that arose during the COVID-19 pandemics, including the WHO ones, stipulating that the amount of aerosols produced during dental procedures should be lowered [16–20, 34, 35].

### Limitations

Regarding the demographic data of our population, our study, based on a self-administered questionnaire, had limitations. First, the participation might have been prone to self-selection bias, and one can hypothesize that dentists who felt concerned with hygiene and prevention measures were more likely to participate. Therefore, and adding to this the limited response rate, they might not precisely represent the entire population of French dentists. Second, our study population was slightly younger than the national population of dentists, with 68% of the respondents being less than 46 years, whereas the mean age of dentists in France is 45 [36]. This was likely due to the delivery mode of the questionnaire via an online popular social media platform. We also observed a majority of women (65%) among the participating subjects, whereas the general population of dentists in France only counts 48% of women [36]. Nevertheless, there is a general tendency toward feminization – with currently more than a half of dental students being female – and towards a rejuvenation of the dentists population in France. Considering the fact that our population was similar to the national population of dentists regarding the type of practice [36], our results could be considered, with caution, as those of the professional population of tomorrow.

With the precautions that have been mentioned before, these results constitute interesting avenues for public health deliberation, which would make it possible to best adapt future health recommendations in order to define the “new normal” of hygiene measures in dental practices at the end of the COVID-19 epidemic.

## Conclusion

The COVID-19 epidemic reshaped the relationship to hygiene and protection measures in the context of dental practices. French dentists have become more aware of the infectious risk in the exercise of their profession, by declaring that they want to maintain, regardless of the sanitary evolution, some of the transitional additional measures enacted in the specific context of the epidemic. The “new normal” of hygiene measures at the end of the COVID-19 epidemic will probably involve more protective measures than before, which will have to find their balance considering, *inter alia*, their acceptance by practitioners.

## Data Availability

The data presented in this study are available on request from the corresponding author.

## List of abbreviations

SARS: CoV-2 Severe acute respiratory syndrome coronavirus 2
COVID: 19 Coronavirus Disease 2019
WHO: World Health Organization
ONCD: Ordre National des Chirurgiens-Dentistes
CNCDUSP: Collège National des Chirugiens-Dentistes Universitaires en Santé Publique
DGS: Direction Générale de la Santé
HAS: Haute Autorité de Santé
PPE: Personal Protective Equipment
INSERM: Institut National de la Santé et de la Recherche Médicale
PPPM: Predictive, Preventive and Personalized Medicine
